# SalmoNet, an integrated network of ten *Salmonella enterica* strains reveals common and distinct pathways to host adaptation

**DOI:** 10.1038/s41540-017-0034-z

**Published:** 2017-10-18

**Authors:** Aline Métris, Padhmanand Sudhakar, David Fazekas, Amanda Demeter, Eszter Ari, Marton Olbei, Priscilla Branchu, Rob A. Kingsley, Jozsef Baranyi, Tamas Korcsmáros

**Affiliations:** 1 Quadram Institute Bioscience, Norwich Research Park, Norwich, NR4 7UA UK; 2 Earlham Institute, Norwich Research Park, Norwich, NR4 7UZ UK; 3 Department of Genetics, Eötvös Loránd University, Pázmány P. s. 1C, H-1117 Budapest, Hungary; 4 Synthetic and Systems Biology Unit, Institute of Biochemistry, Biological Research Centre of the Hungarian Academy of Sciences, Szeged, Hungary; 5 Present Address: Safety and Environmental Assurance Centre, Unilever, Colworth Science Park, Sharnbrook, Bedfordshire UK; 6 IRSD, Université de Toulouse, INSERM, INRA, ENVT, UPS, Toulouse, France

## Abstract

*Salmonella enterica* is a prominent bacterial pathogen with implications on human and animal health. *Salmonella* serovars could be classified as gastro-intestinal or extra-intestinal. Genome-wide comparisons revealed that extra-intestinal strains are closer relatives of gastro-intestinal strains than to each other indicating a parallel evolution of this trait. Given the complexity of the differences, a systems-level comparison could reveal key mechanisms enabling extra-intestinal serovars to cause systemic infections. Accordingly, in this work, we introduce a unique resource, SalmoNet, which combines manual curation, high-throughput data and computational predictions to provide an integrated network for *Salmonella* at the metabolic, transcriptional regulatory and protein-protein interaction levels. SalmoNet provides the networks separately for five gastro-intestinal and five extra-intestinal strains. As a multi-layered, multi-strain database containing experimental data, SalmoNet is the first dedicated network resource for *Salmonella*. It comprehensively contains interactions between proteins encoded in *Salmonella* pathogenicity islands, as well as regulatory mechanisms of metabolic processes with the option to zoom-in and analyze the interactions at specific loci in more detail. Application of SalmoNet is not limited to strain comparisons as it also provides a *Salmonella* resource for biochemical network modeling, host-pathogen interaction studies, drug discovery, experimental validation of novel interactions, uncovering new pathological mechanisms from emergent properties and epidemiological studies. SalmoNet is available at http://salmonet.org.

## Microbiology: SalmoNet-a network resource for Salmonella

A team of researchers led by Tamas Korcsmaros from the Earlham Institute and the Quadram Institute Bioscience situated in UK’s Norwich Research Park have developed a data resource, called SalmoNet, which reveals the internal wiring of the food-borne pathogen *Salmonella*. By integrating experimental and inferred information from three different molecular levels (transcriptional, signaling and metabolic), SalmoNet was generated for 10 different strains of *Salmonella* which could be classified into two phenotypically distinct groups based on their location in the host. SalmoNet not only captures interactions for important *Salmonella* virulence factors but also contains information on the regulation of metabolic processes which are closely linked with disease potential. SalmoNet provides the *Salmonella* community with a resource to broaden and strengthen the ongoing research in network based modeling, molecular pathogenesis and epidemiology, host-pathogen interactions, and drug discovery.

## Introduction

The genus *Salmonella* includes pathogens associated with syndromes ranging from gastroenteritis to bacteraemia and enteric fever.^1^ Gastroenteritis caused by *Salmonella* is one of the most common foodborne diseases, with nearly 100 million cases per year occurring worldwide.^2^ While enteric fever is rare in developed countries, it is still associated with significant mortality and morbidity in low income countries with over 90,000 deaths worldwide in 2015.^3^ *Salmonella* pathogenesis depends on a large number of virulence genes including those located on large pathogenicity islands encoding type III secretion systems that translocate effector proteins into the host cell cytoplasm.^4^ *S. enterica* subspecies includes over 1500 different serovars and accounts for the vast majority of human infections.^5^ Based on the epidemiological record, disease symptoms and observations from experimental infections has resulted in the classification of serovars into two pathovars, namely gastro-intestinal and extra-intestinal. Most serovars of subspecies I are of the former pathovar and most often associated with gastro-intestinal infections. Serovars of gastro-intestinal pathovars often exhibit a broad host range. However, a small number of serovars are host-adapted and are characterized by an extra-intestinal infection and dissemination beyond the intestinal mucosa followed by colonization of systemic sites of the reticuloendothelial system. As most serovars are of the gastro-intestinal pathovar type, the most parsimonious explanation for the extra intestinal serovars is that they evolved from a gastro-intestinal pathovar ancestor, most likely on multiple occasions. The molecular basis of host adaptation has been studied most extensively in *S. enterica* serovar Typhi (*S*. Typhi), the causative agent of typhoid fever. Adaptation of *S*. Typhi is characterized by the acquisition of a number of virulence associated genes and the loss of coding capacity affecting over 200 genes.^6,7^

Genes horizontally acquired by *S*. Typhi include a large pathogenicity island (SP-7) encoding biosynthesis genes for the Vi polysaccharide capsule and the TviA regulator protein,^6^ and the typhoid toxin that is encoded outside SPI-7.^8^ Dissemination beyond the intestinal mucosa is in part mediated by evasion of detection by the host innate immune system by expression of the Vi polysaccharide capsule,^9^ and by the down-regulation of flagella expression, a pathogen associated molecular pattern (PAMP), mediated by TviA.^10^ The function of the typhoid toxin in pathogenesis is not clear, however many of the symptoms of typhoid fever were induced by injection of the typhoid toxin into mice.^11^

However, many of the extra-intestinal serovars of *Salmonella* do not encode SPI-7 or the typhoid toxin. Therefore, alternative mechanisms for the systemic dissemination are likely to have evolved in these serovars. This reflects the convergent evolution to an extra-intestinal lifestyle reflected in the phylogenetic relationship of these pathogens. Convergence in genome sequence polymorphisms of extra-intestinal serovars of *S. enterica* has previously been observed in the form of loss of coding capacity (genome degradation) due to deletions and pseudogene formation.^7^ Degradation of coding sequences corresponding to genes associated with the intestinal phase of infection such as anaerobic metabolism, motility and chemotaxis, and enteropathogenesis was over-represented in these serovars. A similar pattern of genome degradation was also observed in a rapidly evolving hypermutator strain of *S. Enteritidis* that was restricted to a systemic site niche in an immunocompromised patient.^12^

Serovars of *S. enterica* subspecies I exhibit divergence in their nucleotide sequences that corresponds to approximately 737,000 SNPs.^13^ In some cases, non-synonymous SNPs alter the function of proteins, and may alter the function of non-coding sequences when the SNPs are present in regulatory sequences or small RNAs. Serovars also encode hundreds of serovar-specific genes, as well as contain varying degrees of genome degradation that result in considerable differences in coding capacities and gene expression regulation. In light of this complexity, there is a need to apply a systems biology approach to compile network information across the metabolic, transcriptional regulatory and protein-protein interaction (PPI) layers in order to address the hypothesis that extra-intestinal serovars exhibit convergence in molecular mechanisms of host adaptation. Integration of interaction information from multiple layers is expected to provide insights into the shared attributes that characterize *Salmonella* pathogenicity and virulence.

In order to gain further insight into how *Salmonella* host adaptation has evolved there is a need to integrate different levels of knowledge (e.g., metabolism and regulation) as current data resources store the different layers separately, making complex analysis difficult. While a substantial amount of data on regulation, metabolism and protein-protein interactions is available, much of this is curated for model organisms, such as *Escherichia coli*. Integrating different types of information into a complex network remains a challenge for non-model organisms, like *Salmonella*.^14^ For example, in the case of transcriptional regulation, widely used resources such as ORegAnno,^15^ PAZAR^16^, or RegulonDB^17^ do not contain information on *Salmonella*. Other resources such as KEGG^18^ provides information only on metabolic pathways, and even these reactions are not direct *Salmonella* connections but orthology based inferences using *E. coli*. Furthermore, there are no resources that combine curated and predicted interaction information for *Salmonella*. Thus existing resources are either not comprehensive enough to capture multi-layer information or do not contain *Salmonella* specific interaction data.

We therefore compiled the metabolic, regulatory and PPI networks of 10 representative strains of *Salmonella* comprising 5 gastro-intestinal and 5 extra-intestinal strains. In addition to the interactions corresponding to the manually curated information specific to *Salmonella* pathogenicity islands, the networks also contain regulatory interactions derived from high-throughput experiments and whole-genome motif scans apart from interactions inferred from *E. coli* by orthology.

## Results and discussion

### Networks, data representation, and quality control

In this study, we have established a workflow to collect and infer interaction information from three different network levels (metabolic, regulatory and PPI) based on various sources (Table 1). We used data derived from literature, online databases, as well as genome-wide predictions. To further enrich the dataset, we performed this on the whole genome sequence assemblies of a range of *Salmonella* strains (Table 2) representing two pathovars (gastro-intestinal and extra-intestinal) that exhibit distinct life styles. The resulting networks are available to the scientific community for further analysis and enhancement at http://salmonet.org. The networks contain three layers (metabolic, regulatory and PPI) for 5 gastro-intestinal and 5 extra-intestinal strains (Supplementary Table 1).

**Table 1:** Information about the numbers corresponding to the data sources and the reconstructed networks for the reference strain *Salmonella Typhimurium* LT2

Network type	Data source		Number of interactions in *S. Typhimurium* LT2
Metabolic	Model validated by flux-balance analysis^82^		2312
	BioModel database^75^		754
Regulatory	Experimental evidence in *Salmonella*	Manual curation of low-throughput experiments	9
		Datasets containing high-throughput experiments	234
	Genome-wide predictions	Based on experimentally verified binding sites in *Salmonella*	1189
		Based on *E. coli* binding sites from RegulonDB^17^	1865
PPI	Experimental evidence in *Salmonella*	Manual curation	27
		IntAct database^72^	29
	Proteome-wide predictions	Structure based predictions using the Interactome 3D resource^96^	290
		Orthology based predictions using *E. coli* PPI data from,^98^ IntAct^72^ and BioGRID^97^	1846

**Table 2:** Strains included in the study and their life-style

Serovar	Strain	Lifestyle	N.p^a^	Genome assembly ID^b^
Typhi	CT18	Extra-intestinal, causes typhoid fever in humans	2	000195995.1
Paratyphi	ATCC 9150	Extra-intestinal, second most prevalent cause of typhoid fever	0	000011885.1
Choleraesuis	SC-B67	Extra-intestinal, causes swine paratyphoid	2	000008105.1
Dublin	CT 02021853	Extra-intestinal, bovine-adapted serovar	1	000020925.1
Gallinarum	287/91	Extra-intestinal, causative agent of fowl typhoid in poultry	0	000009525.1
Agona	SL483	Gastro-intestinal	1	000020885.1
Enteritidis PT4	P125109	Gastro-intestinal	1	000009505.1
Heidelberg	SL476	Gastro-intestinal	2	000020705.1
Newport	SL254	Gastro-intestinal	2	000016045.1
Typhimurium	SL1344	Gastro-intestinal	3	000210855.2
Typhimurium	LT2	Gastro-intestinal (reference strain closely related to SL1344)	1	000006945.1

^a^ *N.p.* number of plasmids

^b^ GenBank database (http://www.ncbi.nlm.nih.gov/genbank/)

**Fig. 1:**
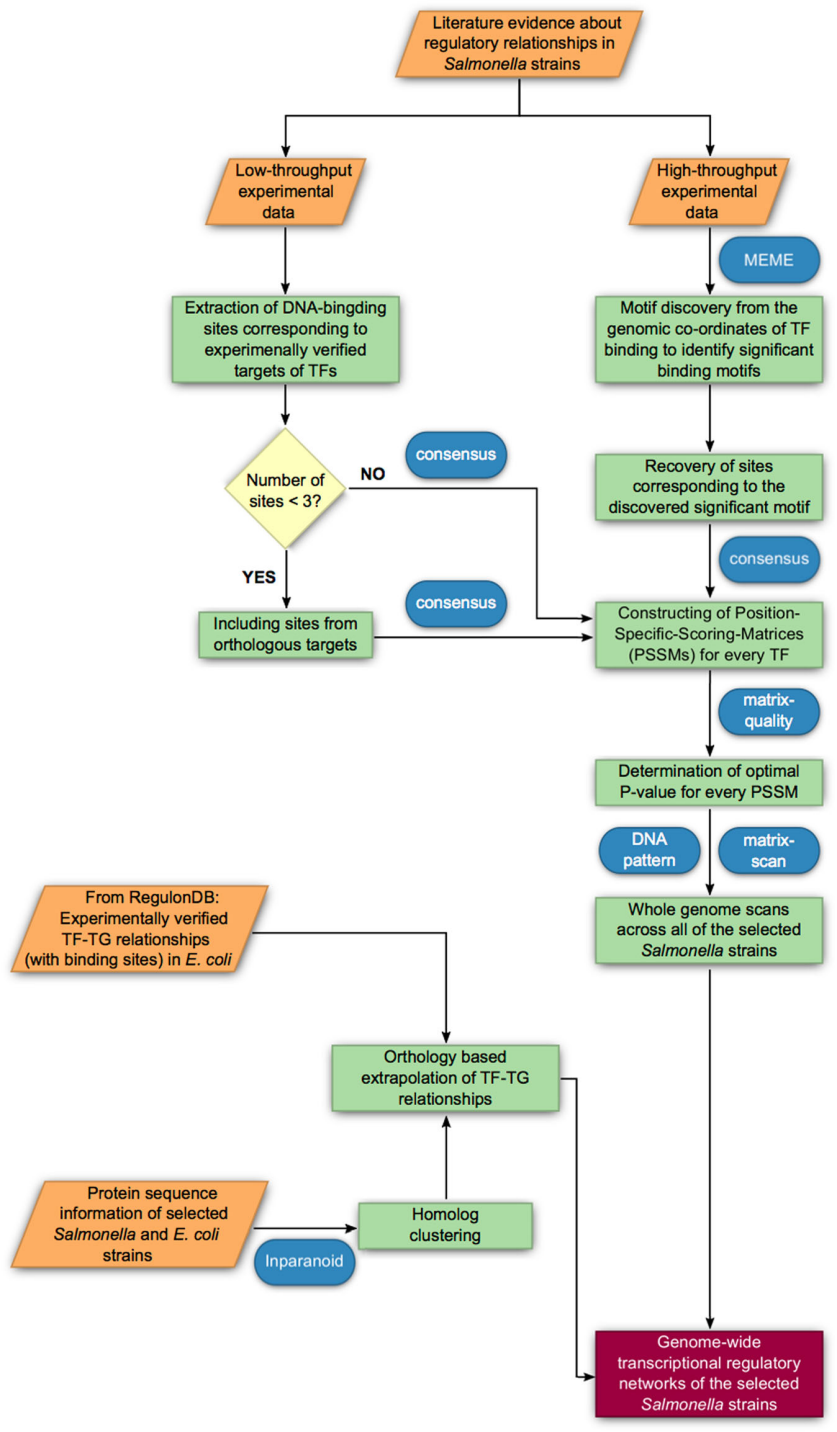
Workflow depicting the steps followed in the reconstruction of the transcriptional regulatory networks of the *Salmonella* strains

The SalmoNet database consists of a total of 81,514 interactions involving 30,870 genes across the studied strains (see the strain specific distribution in Table 3). Considering all the annotated genes for the strains (49,472 genes), SalmoNet therefore covers 62% of the coding capacity of all the strains. In terms of the number of individual ortholog groups, SalmoNet contains information on the interacting partners of 132 sets of transcription factors (TFs) in the regulatory network, 1282 sets of proteins in the PPI network, as well as information on 1196 sets of enzymes in the metabolic network. Of the total 6070 unique connections in the regulatory network, 16% were present in all the 10 strains (Supplementary Figure 1) spanning the gastro-intestinal and extra-intestinal pathovars, thus comprising a core subset of the *Salmonella* regulatory networks inferred from our workflow (Fig. 1). The edges in this core are those that represent higher confidence since they follow the principle of cross-strain conservation.^19^ This methodology has previously been used as a basis for minimizing false positives. The ratio for PPIs and metabolic interactions present in all the 10 strains were higher: 72.6% and 69.2%, respectively. Variation in the fraction of each network represented by the core in regulatory and PPI/metabolic networks supports the idea that transcriptional regulation evolves at a faster rate than the PPI or metabolic levels,^20^ although the noise arising from the heterogeneous sources used for the reconstruction of the regulatory network cannot be ignored. The use of the *matrix-quality* tool^21^ to determine customized *P*-values for every TF-strain combination for the transcriptional regulatory (binding site) predictions minimizes the high false positive rates which could otherwise arise from using generic *P*-value thresholds. Due to the low number of true positive sets, Precision-Recall calculations could not be inferred for most of the transcription factors analyzed in the study. However, to exemplify the reliability of the networks, we determined the target Recall rates (recovery of known targets) for one of the transcription factors SsrB. SsrB regulates the expression of multiple target genes including a number of virulence factors including members of the *Salmonella* pathogenicity islands.^22,23^ 24 instances of the 18 bp SsrB binding motif in *S. Typhimurium* SL1344 have been reported,^23^ By performing a random and equal bifurcation of the known binding sites into test and training sets, we were able to achieve recall rates of 75% in the reconstructed regulatory network for *S. Typhimurium SL1344* (Supplementary Table 2). Furthermore, swapping the test and training sets yielded a recall rate of 64% suggesting that the reconstructed networks are robust in terms of recovery of true positives. With this example, we show that the predicted regulatory interactions in SalmoNet recover previously reported binding sites due to the employed stringencies such as an informed *P*-value. Besides, users can further enhance the networks by choosing only those interactions which occur in multiple strains of each serovar or all the strains in the study depending on their use case.

**Table 3:** Number of genes/proteins and their interactions from the networks for the different *Salmonella* strains

Strain name	Number of proteins	Metabolic network		Regulatory network		PPI network	
		nodes	links	nodes	links	nodes	links
Typhi	4718	1121	2348	1710	2595	1235	1949
Choleraesuis	4607	1137	2390	1650	2913	1223	1953
Dublin	4606	1170	2542	1583	2735	1247	2036
Gallinarum	3943	1140	2432	1484	2628	1200	1924
Paratyphi	4083	1136	2380	1565	2692	1202	1923
Agona	4592	1182	2584	1652	2845	1235	1978
Enteritidis	4192	1206	2653	1680	2921	1266	2062
Heidelberg	4757	1187	2590	1638	2736	1266	2072
Newport	4784	1189	2611	1582	2766	1256	2055
Typhimurium SL1344	4657	1228	2762	1735	3107	1287	2068
Typhimurium LT2	4533	1227	2763	1794	3288	1352	2213
Average	4497	1175	2550	1643	2838	1251	2021

The scientific community can access the database via the aforementioned dedicated web resource in which the molecular entities can be searched by their gene names, UniProt accession IDs, and locus tags. The original source of the interactions that was used during the data integration workflow is also displayed. To enable the comparison of interactions across strains, the ortholog clustering IDs (generated during this study) are also provided for individual molecular entities. An easy-to-use option is provided for users to download selected interaction sets from particular layers of the network or for particular strains. The core *Salmonella* network (the set of interactions conserved across all the strains) can also be accessed similarly. The files are available for download both in CSV and Cytoscape formats allowing straightforward further filtering and visualization, respectively.

### Network dendrograms for comparison among strains

**Fig. 2:**
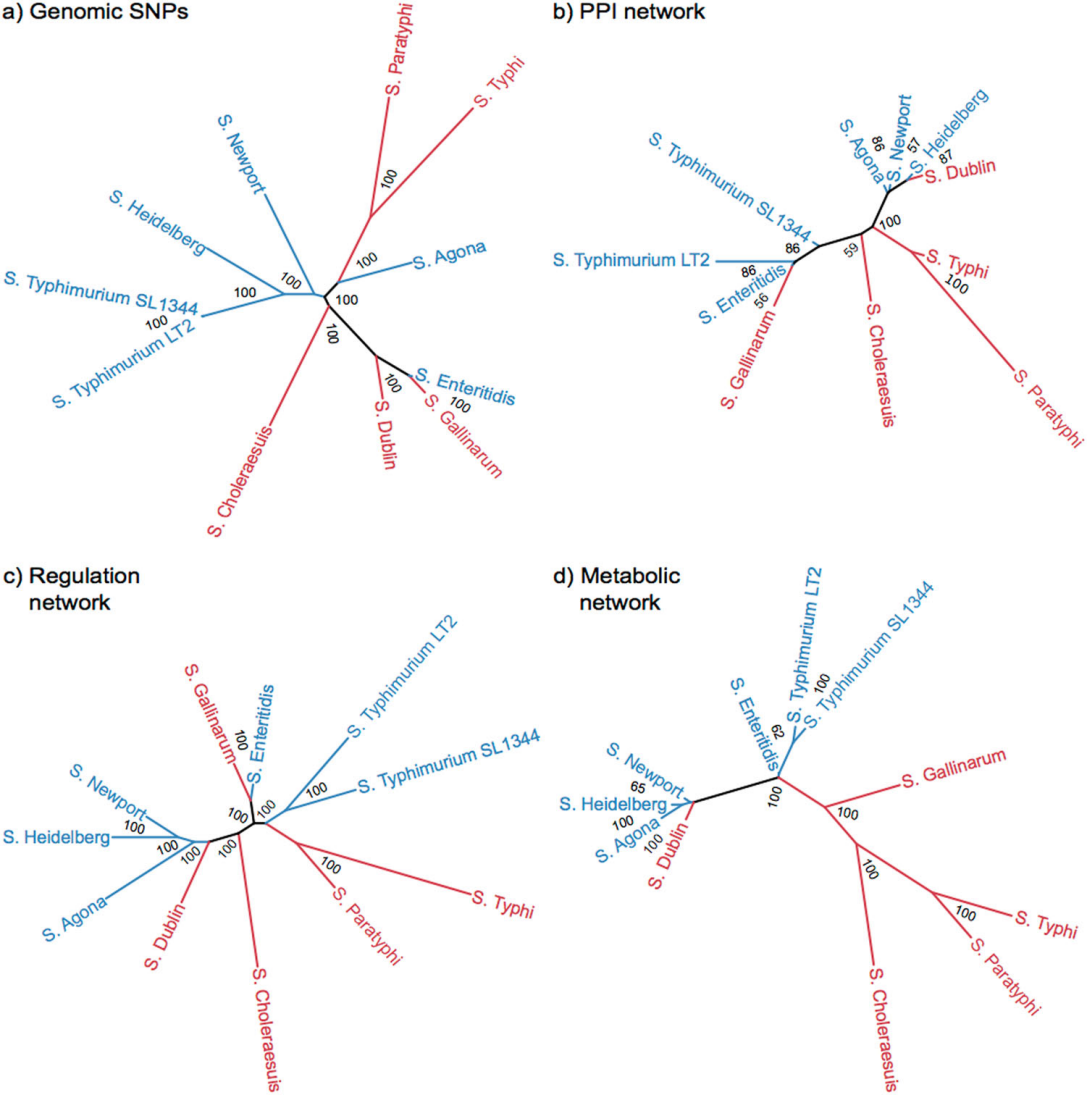
Genome-based phylogenetic tree and hierarchical classification of networks. To distinguish different serovar types, gastro-intestinal serovars were colored to blue and extra-intestinals to red. Posterior probability values (as percentages) are shown on each node. **a** Bayesian phylogenetic tree based on the polymorphic sites of all common genes. **b-d** Hierarchical classification trees based on the matrix representation of protein-protein interaction networks **b**, regulatory networks **c**, and metabolic networks **d**. We note that four strains (Heidelberg, Agona, Newport and Dublin) form a cluster in all the three network based dendrograms due to technical reasons (see details in the main text)

To determine the evolutionary relatedness of the ten *Salmonella* strains in SalmoNet, we constructed a phylogenetic tree based on their nucleotide sequences. All the polymorphic sites found in the common ortholog genes of all the strains were used to build a Bayesian dendrogram (Fig. 2a). Four of the gastro-intestinal strains (*S*. *Typhimurium* LT2, *S*. *Typhimurium* SL1344, *S*. Heidelberg SL476 and *S*. Newport SL254) were monophyletic on the polymorphic site based phylogenetic tree but two of them were clustered together with extra-intestinal strains: *S*. Enteritidis P125109 with *S*. Gallinarum 287 91; and *S*. Agona SL483 with *S*. Typhi CT18 and *S*. Paratyphi A ATCC 9150. The tree was constructed assuming an approximately equal rate of mutation in each strain, and based on this assumption, the common ancestor of these strains is central within the genome based tree. Strains from extra-intestinal and gastro-intestinal serovars could not be distinguished based on the topology of the genome based dendrogram as observed in previous studies.^24^ This is consistent with these pathovars independently emerging as extra-intestinal pathogens and that their attributes arise multiple times during evolution by a process of convergence in genome degradation in the anaerobic metabolism as also described by Nuccio et al.^7^

Next, we compared the phylogenetic relationship of the extra-intestinal and gastro-intestinal pathovars with their metabolic, regulatory and PPI networks to determine if network adaptations converge or if they reflect the evolutionary history of the strains. We used the matrix representation of the networks to infer Bayesian trees corresponding to hierarchical classifications (Fig. 2b–d). The dendrograms for each network were in all cases well established with nearly all posterior probability percentages at the nodes greater than 85. However, none of the networks resulted in the clustering of the extra-intestinal and gastro-intestinal strains. The hierarchical classification based on the metabolic networks separate the two pathovar types most pronouncedly, with only the *S*. Dublin metabolic network exhibiting greater similarity to gastro-intestinal pathovars than extra-intestinal pathovars. This is consistent with the loss of shared metabolic pathways that can be dispensed with by all extra-intestinal pathovars that have in common the loss of intestinal colonization as a mode of pathogenesis. The loss of metabolic pathways associated with the intestinal phase of infection is relatively strongly indicated. There is no evidence for loss of PPIs and regulatory sub-networks in the extra-intestinal pathovars. This could reflect the absence of changes in these networks in response to the dispensing of functions required specifically for the intestinal phase of infection or changes to these networks associated with adaptation to the extra-intestinal environment may be distinct in each extra-intestinal pathovar with weak or absent convergence of mechanisms.

We note that four strains (Heidelberg, Agona, Newport, and Dublin) form a cluster in all the three network based dendrograms (see details in the main text). This is most likely due to the absence of particular genes having interactions to some key genes not present in the data sources used in our pipeline. As SalmoNet only contains genes with interaction data, if one of the interacting pair is missing, and the other interactor has no other interactions, SalmoNet does not contain that particular gene. For these four strains, this methodological limitation resulted in leaving out 31 genes, and because of that, these strains were clustered together.

### Functional enrichment analysis of regulons point to pathovar specific transcription factor functions

**Fig. 3:**
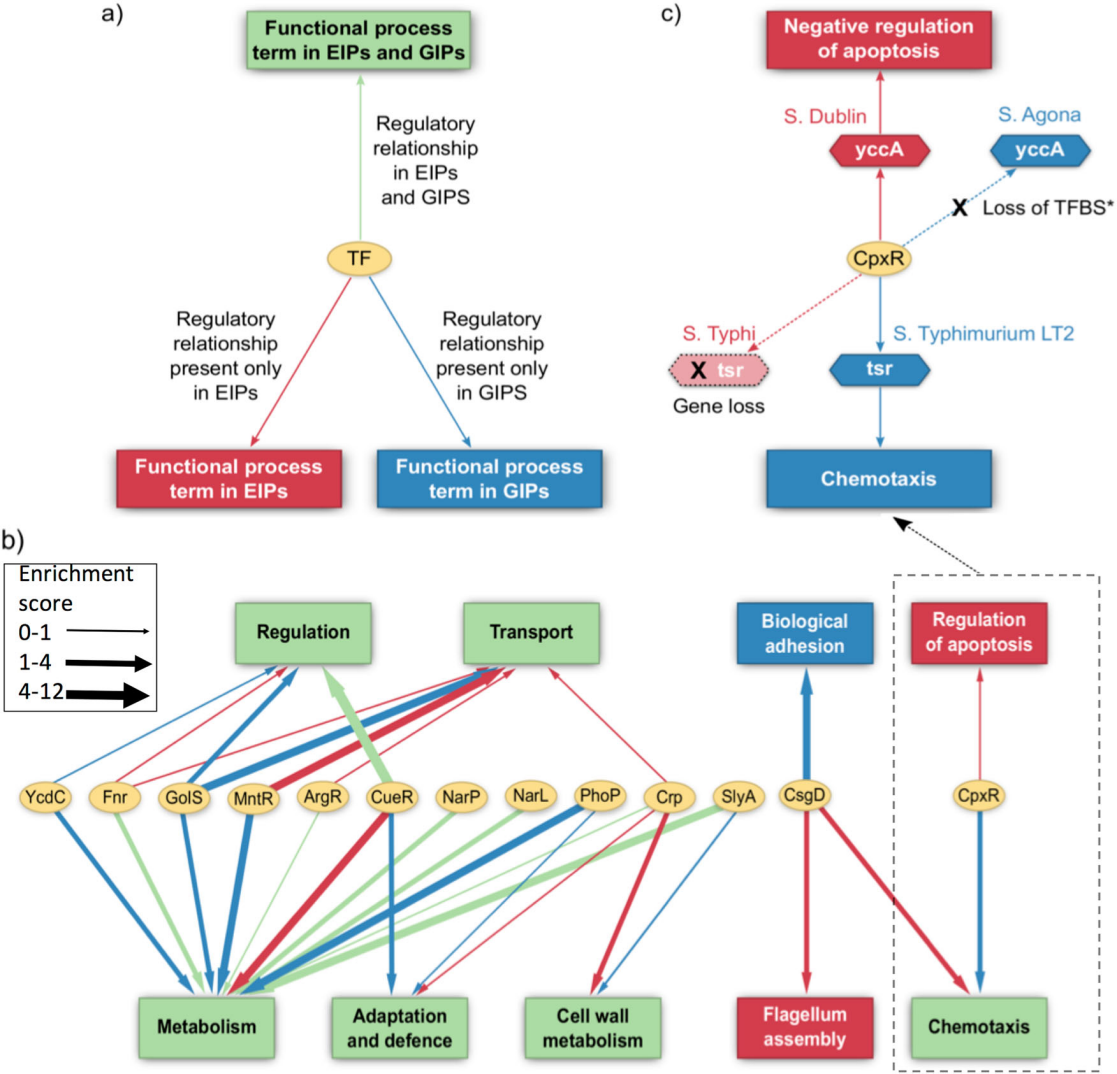
Network of pathovar specific enriched functions and transcription factors. **a** Network legend for the figure. **b** Graphical representation of the functional processes predicted to be commonly and differentially modulated by orthologous transcription factors (TFs) in extra-intestinal and gastro-intestinal pathovars. **c** A specific example, enlarged from **b** demonstrating the loss in gastro-intestinal and extra-intestinal pathovars of regulatory relationships between cpxR and genes involved in the negative regulation of apoptosis and chemotaxis respectively. *TF* transcription factor, *TFBS* transcription factor binding site

Host adapted extra-intestinal pathovars are exposed to distinct host environments and conditions compared to the gastro-intestinal counterparts which are confined to the environment of the intestinal lumen and mucosa. Evolution to this alternative lifestyle was likely accompanied by plasticity in the regulation of functions in the extra-intestinal pathovars. Extra-intestinal pathovars mediating systemic infections are associated with increased severity and distinct pathogenicity.^25–29^ Enrichment analysis of the putative regulons revealed regulation of different functional processes in the two pathovar types by the same orthologous transcription factor (Fig. 3a–b). For instance, the virulence modulating regulator (CsgD) is known to control the expression of various pathogenicity related genes which are important for virulence, persistence and biofilm formation.^30,31^ In our analysis, the set of genes putatively regulated by CsgD were found to be enriched with the specific functional process of ‘Biological adhesion’ in gastro-intestinal pathovars. However, the functional process of ‘Flagellum assembly’ was over-represented among the putative CsgD targets in extra-intestinal pathovars while ‘Chemotaxis’ was over-represented in both extra-intestinal and gastro-intestinal pathovars representing specific differences and commonalities in the role of CsgD between the two pathovars. Similarly, comparative analysis of the CpxR regulons revealed pathovar-specific enrichment of functions within the regulons. CpxR encodes a cognate response regulator and forms part of the CpxAR two component system involved in the sensing of and response to various cell envelope stresses and stimuli.^32,33^ In accordance with already known information that CpxR regulates motility and chemotaxis,^34^ the set of putative targets of the CpxR regulon in gastro-intestinal pathovars was enriched with the functional process of chemotaxis. In the extra-intestinal pathovars, however, the functional process of regulation of apoptosis was found to be over-represented as a result of distinct cis-regulatory differences. For example, the gene encoding the YccA protein in the extra-intestinal serovar harbored a CpxR binding site in its promoter region while the YccA ortholog in the gastro-intestinal serovar was observed to have a complete loss of the CpxR binding site due to truncation of its promoter (Supplementary Figure 2, Fig. 3c). YccA is homologous to the human anti-apoptotic protein BI-1 which represses the activity of the tumor suppressor protein Bax.^35^ Due to the high conservation between *E. coli* YccA and the human BI-1, the YccA protein was even able to protect yeast cells against apoptosis induced by ectopically expressed human Bax protein^36^ thus suggesting that YccA is associated with the modulation of host apoptosis. Other apoptotic related factors regulated by CpxR include genes or their orthologs encoding proteins such as the periplasmic serine endoprotease DegP/HtrA,^37^ Hemolysin expression-modulating protein Hha,^38^ and the toxicity modulator TomB with which Hha forms a putative toxin–antitoxin pair.^39^ CpxR also modulates the expression of members of two distinct classes of proteins namely porins (such OmpF) and chemotaxis related proteins (such as CheA, CheW) which are known to modulate apoptosis in the host cell upon infection.^40,41^

Apoptosis of macrophages is a common host response once Salmonella has established an infection systemically but this is tightly regulated as the over-induction of apoptosis is detrimental to Salmonella.^42^ Hence, given that extra-intestinal pathovars cause systemic infections, it may be beneficial for the extra-intestinal pathovars to down-regulate apoptosis. This is one possible explanation for the over-representation of apoptosis regulation genes within the CpxR regulon in extra-intestinal pathovars and could indicate that CpxR plays a role in modulating apoptosis in extra-intestinal pathovars. The importance of CpxR in extra-intestinal pathovars is also demonstrated by studies which point out the use of CpxR as a safe and effective vaccine candidate against fowl typhoid caused by *Salmonella* Gallinarum, an extra-intestinal serovar. The results from the compositional analysis of the regulons indicate that the regulons of the two pathovars may have evolved to adapt to their respective pathogenic niches. The differences with respect to the functional processes regulated by *Salmonella* transcription factors could essentially be due to the expected adaptive evolution of extra-intestinal pathovars in contrast to the gastro-intestinal pathovars, which are mostly confined to the gut.

### Applications of the database

The molecular interactions forming the biological interface between *Salmonella* and its host play a significant role in the colonization and infection process. *Salmonella* pathogenesis depends on its ability to adhere to host epithelial cells and the Type III secretion system mediated injection of effector proteins, which then cause the re-arrangement of the host cell cytoskeleton and internalization.^43–46^ *Salmonella* resides, survives and multiplies in specialized membrane bound vacuoles.^47,48^ Various genes known to be involved in *Salmonella* virulence and pathogenesis have been implicated in the interactions of *Salmonella* with the host cells.^43–46^ From the regulatory networks in SalmoNet, the transcription factors, which potentially regulate the virulence genes whose products are involved in the interactions with the host, can be identified. Moreover, by merging the regulatory networks with an increasing number of datasets such as the PPIs between *Salmonella* and the human host,^49^ it is expected to enhance our understanding of the increasing number of mechanisms employed by *Salmonella* to infect and survive inside host cells.

Although the PPI and metabolic networks were inferred by orthological extrapolation, the original sources of data from which the extrapolation was performed are reliable due to their experimental basis even though the source data for the PPI networks were derived from high-throughput techniques. Hence, given the lack of PPI data for these *Salmonella* strains in this study, the inferred PPI and metabolic networks can be considered as a primary starting point for hypothesizing and uncovering new mechanisms. The PPIs are very interesting in this regard since previous studies have shown that *Salmonella* modulates many post-translational modifications such as ubiquitination of host proteins in order to avoid host responses such as autophagy.^50^ The multi-layered nature of the SalmoNet resource can also be exploited in order to uncover potential biological insights by which *Salmonella* subverts host mechanisms. Recent evidence points to the modulation of metabolism (both of the *Salmonella* and the host) as yet another mechanism employed by *Salmonella* to acquire nutrients, evade host defense and survive under harsh intracellular conditions.^51–59^

An integrative analysis of the regulatory and metabolic networks has the potential to reveal new insights into the transcriptional regulatory modulation of metabolic enzymes and could identify new metabolic drug targets as an intervention strategy. Integrating the PPI and regulatory networks not only provides a combined view of signal transduction and gene regulation but also help us to shortlist upstream regulators of genes involved in establishing infection and metabolic functions. The activities of such individual regulators and transcription factors can be taken up for testing and screened for inhibition by small molecules/antibiotics.^60^ Besides, SalmoNet also provides information on binding sites which can be used to design transcription factor decoys (anti-sense nucleotides of the transcription factor binding motif)^61^ that prevent the binding of transcription factors to their targets. Anti-sense oligonucleotides have been used to modulate gene expression in a wide variety of intracellular bacterial pathogens such as *E. coli*,^62,63^ *Salmonella* *Typhimurium*,^62^ *Enterococcus faecalis*,^64^ and *Klebsiella pneumoniae*.^65^ Clinical trials have also been performed using anti-sense oligonucleotides for the treatment of human diseases^66,67^ thus suggesting that the potential of using anti-sense oligonucleotides against bacterial infections looks promising. In addition, the proteins and enzymes involved in critical PPIs and metabolic reactions respectively can also be subjected to the classical or systems-based drug-discovery pipelines.^68^ The multi-layered network of SalmoNet allows discovering new molecular targets and strategies for therapeutic or prophylactic interventions based on the emergent properties of the networks. Modern drug and target discovery pipelines^69,70^ advocate a systemic approach, which relies on the integration of various heterogeneous data such as expression profiles and other multiple prior knowledge networks. SalmoNet satisfies this need by providing the prior knowledge networks for various strains of *Salmonella*.

As a source of both experimental and predicted interactions, SalmoNet contains information on the transcriptional regulatory targets of various transcription factors based on genome-wide motif scans. In addition, predicted targets of recently characterized transcription factors, such as RtsB,^70^ which regulates the expression of invasion and flagellar genes, are also provided for future experimental verification and validation. Similar experimental testing and verification can also be performed on the predicted PPIs as well given the importance of PPIs in the survival of *Salmonella* inside the host cells.

From an epidemiological perspective, information on networks of multiple strains and strain-specific interactions further enriches *Salmonella* epidemiology studies. Traditional epidemiological approaches rely on tracking genetic polymorphisms and loss/gain of virulence genes specific to certain contexts and conditions. Hence, interaction networks could help to evaluate the effects of genetic polymorphisms in a systematic way, and thus, help in filling the gap between observed phenotypes of mutated strains and their genotypes. For example, SalmoNet can be used to further investigate the effect of cis-regulatory mutations on interactions, as well as the network level properties which determine the virulence characteristics of different strains of *Salmonella*.

### Benchmarking *Salmonella* network data

There is no single resource storing *Salmonella*-specific protein-protein interactions (PPIs) but they are captured in general databases such as STRING^71^ and IntAct.^72^ In STRING, PPIs are based on different types of data such as genomic context, co-occurrence, co-expression, data derived text-mining and imported data from IntAct and other PPI resources. STRING contains only 237 experimentally verified interactions in addition to 1014634 predicted interactions based on criteria such as neighborhood, gene-fusion, gene-co-occurence, co-expression, and text mining among *Salmonella* proteins. In IntAct, which contains manually curated and also imported PPI data from other databases, there are only 31 PPIs for *Salmonella* proteins.

In the case of transcriptional regulatory networks, RegulonDB^17^ stands out as one of the most comprehensive repositories for prokaryotic gene regulation. However, RegulonDB is restricted to *E. coli*. RegPrecise^73^ contains information for multiple bacterial species using genome-wide predictions based on manually curated reconstruction of regulons (which are set of genes whose expression is regulated by a transcription factor). Unfortunately, RegPrecise does not provide the original source of data used for the predictions, making further application of the data difficult. While well-known sources such as ORegAnno^15^ and PAZAR^16^ also capture regulatory interactions for multiple species, they do not contain any interactions for *Salmonella*.

As for the metabolic networks, there are numerous resources such as KEGG,^18^ MetaCyc/BioCyc^74^ and the BioModels databases^75^ containing seemingly *Salmonella* specific metabolic reactions. However, these databases are either not curated systematically or are not based on experimental results. KEGG for example contains information on pathway reactions and their entities for a large number of *Salmonella* strains but the *Salmonella* pathway annotations are based on computational predictions and not on experimental data. Similarly, coliBASE^76^ captures comparative genomic data in terms of whole genome alignments and ortholog gene lists for *Salmonella*. Further information on bacterial metabolism can be found in specialized databases such as PATRIC for pathogens^77^ or TRACTOR DB for Gamma proteobacteria.^78^ However, most of the above mentioned resources contain limited interaction information for *Salmonella* and do not enable researchers for comparative network analysis or systems biological modeling of processes other than metabolism (e.g., they do not provide regulators of metabolic processes).

## Conclusion

We present the first public biological network resource for *Salmonella* research. SalmoNet contains network data (metabolic, regulatory, protein-protein interaction) for 10 representative pathogenic *Salmonella* strains. To elucidate the virulence program of *Salmonella* for either discovering knowledge on biological mechanisms or for therapeutic interventions, it is rewarding to integrate the different network layers that capture emergent properties of the system. SalmoNet represents a resource which contains information on interactions from multiple layers of biological organization that can be analyzed as such, or as a topological backbone to be integrated with new -omic datasets for analyzing the dynamics. SalmoNet opens the possibility for systems-level studies of the pathogen *Salmonella* with unprecedented details in a standardized and well documented format. The resource can be browsed and downloaded as a whole or in user-defined interaction sets at http://salmonet.org. The analysis of SalmoNet could go far beyond fundamental biological and systems biology research. SalmoNet can be applied by medical microbiologists and epidemiologists to understand the strain specific differences of *Salmonella* and can serve as a starting point for further experimental investigations and systems medicine based drug discovery.

## Methods

### Strains and orthology

Five strains of serovars with a predominantly gastro-intestinal lifestyle of *Salmonella* and five strains of serovars of extra-intestinal lifestyle were selected (Table 2). We included *Salmonella enterica subsp. enterica serovar Typhimurium* str. SL1344 as a sixth gastro-intestinal strain since it is widely used as a reference strain. We determined orthologous proteins among the selected strains, as well as for the model organism *E. coli* K12 with InParanoid.^79^ We used a reciprocal best hit approach using BLAST to identify homologous protein sequences including those from plasmids corresponding to the selected strains. The protein sequences were downloaded from UniProt^80^ as of January 2015. The results from the comparison of proteins one by one among any pair of strains were used to derive the ortholog clusters. Sequence similarity was set at > = 95% in order to minimize false positives given that the chosen strains belonged to the same species. Clustered groups contained both paralogs and orthologs (Supplementary Table 3). We removed the pseudo-genes listed in ^7^ from the resultant ortholog list.

### Reconstruction of networks

We developed metabolic, regulatory and protein-protein interaction networks for *Salmonella*, using complementary approaches followed by merging the three layers into a unified *Salmonella* network. We performed this process for the 10 strains separately that resulted in 10 strain specific molecular networks.

Metabolic networks: We defined the metabolic network as follows: if a metabolite is a product of a reaction and substrate in another, the two proteins catalysing the different reactions were linked, as described in ref. 81 We did not consider the links for metabolites appearing in more than 10 reactions as outlined in ref. 81

We retrieved the metabolic reactions from two different sources with different levels of curation: the manually curated metabolic model of *S*. *Typhimurium* LT2 (referred to as STM),^82^ and predictions from the BioModels database^75^ containing Enzyme Commission (EC) numbers. In the latter, EC number assignments are automatic and include predictions for enzymes present in *Salmonella spp* and not necessarily present in *E. coli*. The STM model was derived from an *E. coli* metabolic model and confirmed by flux balance analysis. For the extrapolation of metabolic reactions from the above mentioned models, we assumed that the same reactions occur in the *Salmonella* strains when orthologous protein(s) of the enzyme(s) involved in the reactions were found to be present in the corresponding strains.

Regulatory networks: Regulatory interactions represent the binding of transcription factors to gene promoters. They consist of both predicted and experimentally verified interactions in our study. We collected experimentally verified DNA-binding sites of *Salmonella* transcription factors (Supplementary Table 4) from the literature with manual curation, as well as information from publicly available datasets. For the high-throughput datasets retrieved from,^83^ peaks were identified as described elsewhere.^84,85^ For all the other high-throughput datasets, the processed data (transcription factors, their targets and corresponding binding motifs when available) was retrieved from the cited sources (Supplementary Table 4). We then constructed Position Specific Scoring Matrices (PSSMs) from the manually inferred binding sites and sites corresponding to the significant consensus motifs from the low- and high-throughput datasets respectively using the *consensus* tool^86^ with default parameters. PSSMs constructed from too few binding sites have low predictive values. Hence, in instances where the number of binding sites (according to published data) corresponding to a transcription factor were less than three, we used corresponding sites from orthologous targets present in one or more of the other *Salmonella* strains under study for the PSSM construction. Since the predictive capacity and information content varies among PSSMs, we determined specific optimal P-value thresholds for every PSSM-strain combination using the *matrix-quality* tool^21^ (Supplementary Table 5). We used the TRANSFAC-formatted PSSMs via the *matrix-scan* tool^87^ to scan the promoter regions of all the genes from the genomes of the selected *Salmonella* strains. We confined the binding site search to 5000 bp upstream of the start codon of every protein coding gene to capture functionally active transcription factor binding sites in genomic regions including intergenic sequences between convergent genes.^88^ However, sequences which overlap with upstream coding sequences were excluded. The promoters were retrieved using the “retrieve sequence” function of the RSAT tool suite. For the background model, we used a Markov order of 1, and the model was estimated individually for every strain. Both the strands of the genome were scanned for the presence of putative binding sites and the optimal P-value determined for every TF-strain combination as described previously was used during the corresponding scans. Putative hits with a *P*-value lower than the corresponding optimal cut-off values were considered to be significant. Based on the principle of “regulogs”,^89,90^ we also inferred transcription factor-target gene relationships in *Salmonella* strains. Regulogs are regulatory interactions first detected in one species (in this case in *E. coli*) and then predicted to be potentially present in another species (in this case in *Salmonella*) based on sequence homology of the transcription factor, the target gene and the transcription factor binding site. Accordingly, we used the *E. coli* transcription factor - target gene binding site information retrieved from RegulonDB^17^ in conjunction with the homolog clustering results to extrapolate regulatory interactions from *E. coli* to the *Salmonella* strains. Operon information was retrieved from DOOR.^91^ The workflow is presented in Fig. 1.

PPI networks: We performed manual curation to retrieve existing PPI information in *Salmonella* spp from the literature using a curation protocol we developed for the SignaLink eukaryotic signaling network resource as previously described.^92,93^ Briefly, we collected signaling interactions involving *Salmonella* proteins from primary research articles identified by using iHOP^94^ and ChilliBot^95^ tools in addition to those articles directly found in PubMed searches. The main text and the abstracts of these articles were examined to retrieve the interactions between *Salmonella* proteins. Experimentally verified *Salmonella* PPIs were retrieved from IntAct.^72^ Proteome-wide predictions to predict PPIs were carried out using 3D protein-based structure predictions of Interactome 3D^96^ and using *E. coli* PPIs for interolog predictions. The interologs were inferred based on *E. coli* PPIs retrieved from IntAct,^72^ BioGrid^97^ and a high-throughput, yeast-2-hybrid screen of the *E. coli* interactome.^98^

### Phylogenetic tree construction

Gene sequences corresponding to the *Salmonella* strains considered in this study were downloaded using the *retrieve-sequence* tool from the Regulatory Sequence Analysis Tools.^87^ Out of the 2912 common ortholog gene sets from the strains in this study, 457 ortholog sets were discarded due to discrepancies such as misconverted locus tags/IDs. Ortholog genes that had different lengths across strains were aligned by using ClustalOmega^99^ implemented in the *msa* Bioconductor R package.^100^ We identified 85 ortholog gene sets where one or more strains had more than one sequences (due to gene duplication or misannotation). We discarded these extra sequences after manual curation and the sequences that were more similar to the sequences of other strains were retained.

MrBayes v3.2.4^101^—which is a program for Bayesian inference based selection of evolutionary models—was used to analyze the phylogenetiic relationships of the strains using the polymorphic sites from genes in the orthologous gene sets. The parameters of the evolutionary model between the sequence sites were unlinked. Gaps were not considered as polymorphisms since the applied phylogenetic software treated them as missing data. Thus, gaps did not contribute to the phylogenetic information. Ortholog groups whose ratio of polymorphic sites to gene lengths was more than 0.1 (100 genes) were discarded and consequently polymorphic sites from potentially *false* orthologs that had low sequence similarity were excluded. After applying the above filter, 64,531 polymorphic sites from 2360 orthologous genes were used to infer a genome based phylogenetic tree. Metropolis coupling Markov Chain Monte Carlo (MC^3^) analysis was performed for 10 million generations and 25% of the samples from the beginning of the chain were discarded when applying MrBayes.

### Network based dendrograms

The networks (Supplementary Table 6) were represented by interaction matrices containing binary data, where “1”-s represented the presence of an interaction between the same pair of nodes inferred by orthology across the strains and “0”-s stood for missing interactions (Supplementary Table 6). In order to represent the hierarchical classification of strains from network data, we constructed dendrograms based on the metabolic, regulatory and PPI interaction matrices using MrBayes v3.2.4. To calculate network based dendrograms, the same MrBayes MC^3^ analyses were performed as for the genome-based tree except that the datatype was set to “restriction” and no substitution model was applied.

### Functional analysis of the transcriptional regulatory network

In order to understand the biological context within the regulons of the two serovars, functional enrichment analysis was performed to infer the over-represented Gene Ontology (GO) Biological Process Terms within the predicted regulons. Here, we considered only those interactions that were predicted to occur in at least two of the ten studied strains. This was performed to minimize the chances of including possible false positives in our analyses. In addition, we considered only the predicted regulons and GO terms containing at least 10 entities across all the strains within each pathovar. The background set comprised the entire collection of genes with annotated GO terms in the genomes. To determine the enriched GO Biological Process terms, the hypergeometric test with the Bonferroni correction was applied. The significance score for each enrichment event was calculated as the -log10 function of the corrected *P*-value. Enriched terms with a significance score greater than zero were considered as significant. We retrieved TF-GO relationships, which were exclusive to either of the two serovars. We restricted the analysis to TFs that were predicted to contain different enriched GO Biological Process terms within their putative regulons in extra-intestinal and gastro-intestinal pathovars. We also performed a manual assignment of functional processes derived from the Gene Ontology database for every GO term identified in the previous step. Subsequently, we replaced GO terms with their corresponding functional process(es) in order to simplify the graph without losing information.

### Data availability statement

The datasets generated in the study are freely available at http://salmonet.org/. The source data as well as the generated datasets are provided as Supplementary tables which are freely available via *NPJ Systems Biology and Applications website*. The tools and resources such as the RSAT suite, Mr. Bayes, InParanoid, iHOP, ChilliBot, ClustaOmega, RegulonDB, Interactome 3D, IntAct, BioGrid, and UniProt which are used in this study are publicly available. Custom codes used in the study are available upon request.

## Electronic supplementary material

Supplementary Figures

SI table 1

SI table 2

SI table 3

SI table 4

SI table 5

SI table 6
