# Supplementary material for: SalmoNet, an integrated network of ten Salmonella enterica strains reveals common and distinct pathways to host adaptation
Source: NPJ Syst Biol Appl. 2017 Oct 18;3:31. doi: 10.1038/s41540-017-0034-z (PMC5647365; doi:10.1038/s41540-017-0034-z)
Supplement: Supplementary file 1 — Supplementary Figures [file 41540_2017_34_MOESM1_ESM.docx]

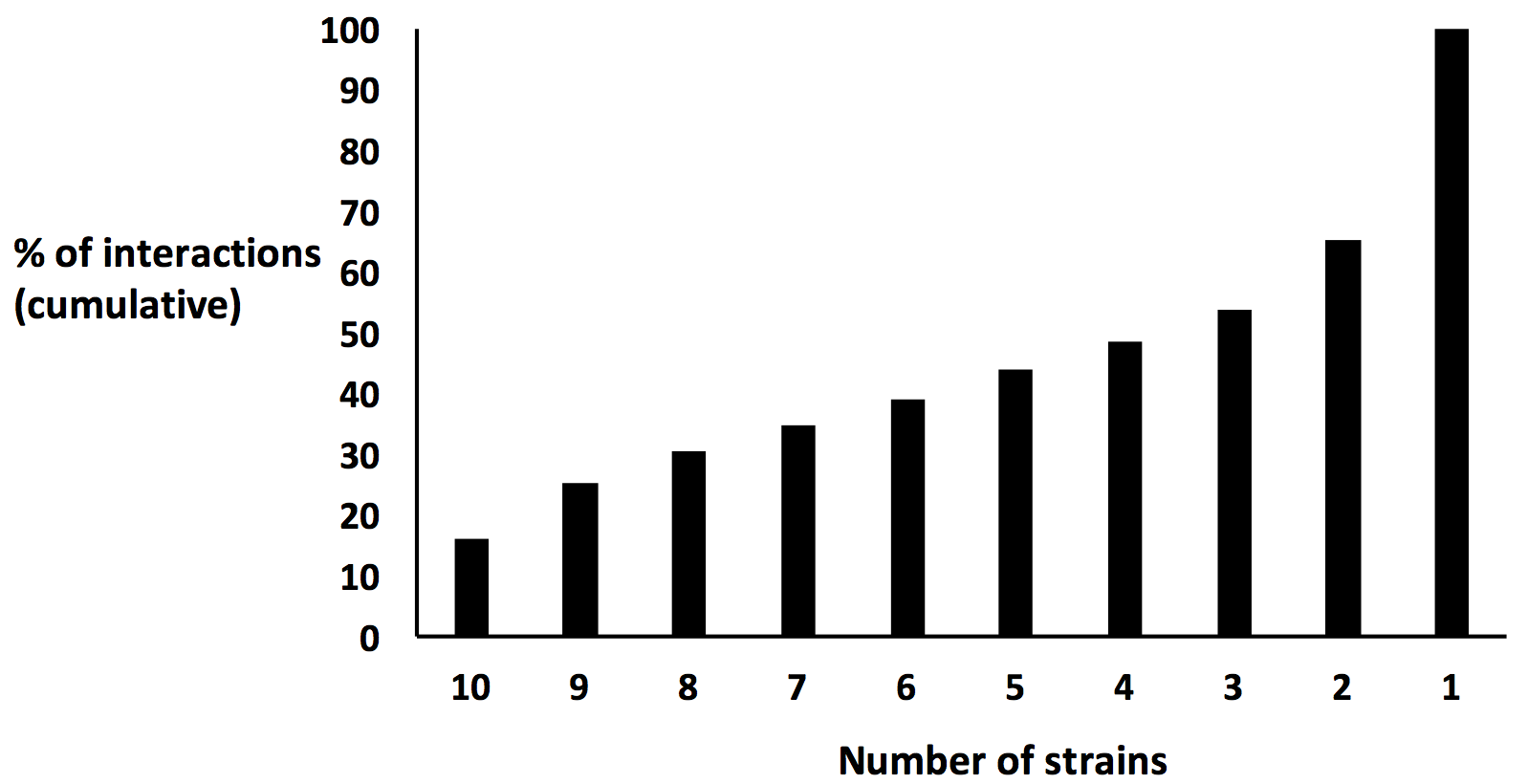


**Supplementary Figure 1:** Histogram showing the fraction of all of interactions present in varying number of strains.


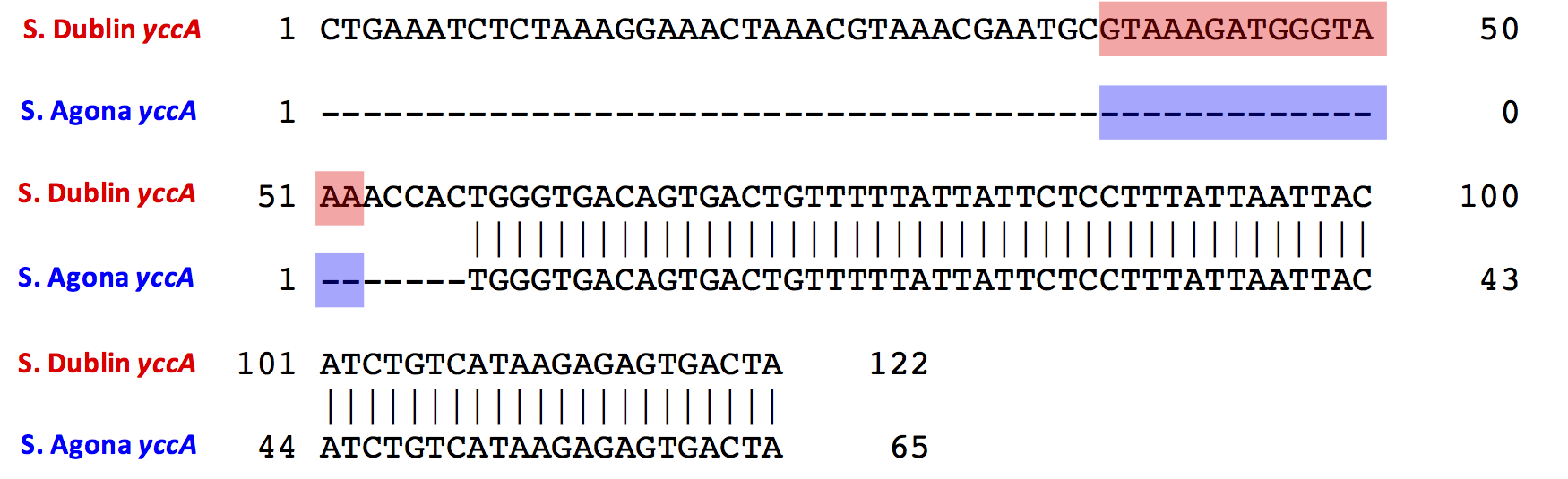


**Supplementary Figure 2:** Figure showing the allignment of the S.Dublin and S.Agona *yccA* promoters as well as the truncation of the CpxR binding site in S. Agona. The EMBOSS Needle tool with default parameters was used to build the allignment.
